# Prevalence and correlates of bullying in physiotherapy education in Nigeria

**DOI:** 10.1186/s12909-020-02019-2

**Published:** 2020-04-15

**Authors:** Chidozie Emmanuel Mbada, Idowu Phebean Ogunseun, Francis Oluwafunso Fasuyi, Oluwafemi David Adegbemigun, Clara Toyin Fatoye, Opeyemi Ayodiipo Idowu, Olubusola Esther Johnson, Adesola Christiana Odole, Adaobi Margaret Okonji, Bashir Kaka, Francis Fatoye

**Affiliations:** 1 Department of Medical Rehabilitation, College of Health Sciences, Obafemi Awolowo University, Ile-Ife, Nigeria; 2 Department of Physiotherapy, Faculty of Allied Health Sciences, University of Medical Sciences, Ondo, Nigeria; 3 Department of Health Professions, Faculty of Health, Psychology and Social Care, Manchester Metropolitan University, Manchester, UK; 4 Department of Physiotherapy, School of Basic Medical Sciences, College of Medical Sciences, University of Benin, Benin City, Nigeria; 5 Department of Physiotherapy, Faculty of Clinical Sciences, College of Medicine, University of Ibadan, Ibadan, Nigeria; 6 Department of Physiotherapy, Faculty of Allied Health Sciences, Bayero University, Kano, Nigeria

**Keywords:** Bullying, Physical therapy, Modalities, Specialty, Nigeria, Students

## Abstract

**Background:**

Bullying is an unexpressed part and parcel of medical education but it is largely unexplored in physiotherapy. This study assessed the prevalence and socio-demographic correlates of bullying in physiotherapy education in Nigeria.

**Methods:**

Two hundred and nineteen clinical physiotherapy students from three purposively selected Federal Universities in Nigeria participated in this study. Following a cross-sectional design, the Students Perception of Professor Bullying Questionnaire (SPPBQ) was used to obtain information on bullying. The SPPBQ includes a working definition of lecturer bullying followed by other sections inquiring about lecturers bullying experiences. Data was collected on socio-demographic characteristics, bullying experiences and availability of adequate policy and support on bullying. Descriptive and inferential statistics were used analyze data. Alpha level was set at *p* < 0.05.

**Results:**

Lifetime and point prevalence of bullying in physiotherapy education were 98.6 and 99.1%. 94.5% of the respondents had witnessed physiotherapy students bullying and there was a 100% rate of ‘no attempt’ to stop a physiotherapy lecturer from bullying. 38.4 and 44.7% of the respondents believed there was adequate school policy and support available on bullying. There was no significant association between bullying and each of age (휒2 = 0.117, *p* = 0.943), gender (휒2 = 0.001, *p* = 0.974), level of study (휒2 = 0.000, *p* = 0.995) and any specific university (휒2 = 1.343, *p* = 0.511).

**Conclusion:**

There is high lifetime and point prevalence of bullying in physiotherapy education in Nigeria, which are largely unchallenged or redressed. Being a clinical physiotherapy student ordinarily predisposes to bullying without necessary contributions of intrinsic and extrinsic factors.

## Background

Bullying is described as the misuse of power or position to persistently criticize and condemn; to openly humiliate and undermine an individual’s ability until they become so fearful that their confidence crumbles and they lose belief in themselves [1], leaving the victim [s] feeling hurt, vulnerable, angry or powerless [2]. Bullying exists in various forms and in various places. Specifically, workplace bullying is commonplace and diverse in nature [3, 4]. Workplace bullying may involve verbal, physical or psychological act which may be encouraged by imbalance of power between the superiors and the subordinates [5, 6] and in some other instances it may occur between coworkers or from subordinates to superiors [3, 7]. Also, there is substantial literature on occupational bullying in the health sector [8–10], especially, among physicians [11–13], nursing practitioners [14–16], dental practitioners [17] and health care administrators [18].

Similar to the foregoing, bullying in the context of health professions education has been documented. As a type of school bullying, it is often characterized by verbal, physical, sexual or emotional harassment or in some cases, a cyberbullying [19, 20]. The United Nations Educational, Scientific and Cultural Organization (UNESCO) [21] submits that gender norms, social norms and the peculiarity of the context underlie school bullying. Specifically, discriminatory gender norms promote male dominance and the suppression of women; while social norms legitimize the authority of lecturers over students [21]. Other authors have documented some other sociodemographic factors such as religion, race and culture to independently or in association promote school bullying [22–24].

Rautio et al. [25] assert that the medical student is the worst hit by bullying [25]. Other researchers concur that bullying is one of the critical stressors students in tertiary institutions around the globe face, especially among those training to become health professionals [26–28]. The period of training to become a health professional remains a stressful one; exposing trainees to situations and experiences with appalling implications for their psychological well-being [29]. In this period, while having to put up with the pressure of a demanding and competitive health professional education, many students are harassed and bullied. The disparagement and belligerence that bullying instills may explain the suicidal ideation of some students and account for unprofessional conducts by some health professionals during practice [29]. Though this topic has been in discussion for ages, especially among medical and nursing students [28–31], it is still one of the least prioritized concerns in the education of other health professionals.

Lecturer bullying may have severe consequences for student victims, including negative psychosocial and behavioral outcomes such as loss of trust, feelings of hopelessness and depression, oppositional behavior and increased fighting amongst peers [32]. Therefore, lecturer bullying remains a “delicate issue” [33] and indeed, an extant issue that cannot be denied [34–38]. Research on professor/instructor bullying is important given findings that university students’ perception of rapport with their professors/instructors predicts motivation, perceptions of learning and perceived grades [39]. In all of these, not much is known about bullying in the physiotherapy practice and education. Seager [40] submit that there is very limited research on bullying in the physiotherapy profession, as only one United Kingdom study was found on bullying among physiotherapy students [41]. Thus, the issue of bullying from the perspective of the physiotherapy profession, as well as from the sub-Saharan African context seems to have drawn little or no attention, except for few studies reporting on workplace bullying within the African context [42, 43]. Unfortunately, based on empirical reports, Nigeria, like most other countries in sub-Sahara Africa is notorious for human right abuses [44, 45]. The UNESCO [21] submits that ‘*schools and the education system also operate within the context of wider social and structural factors and may reflect and reproduce environments that do not protect students from violence and bullying’*. Anecdotally, bullying is a common occurrence in the Nigerian setting, which may be suggestive of the pattern in the wider social context. To our knowledge there seems to be no local unpublished research reports which highlight this knowledge gap. Therefore, this study assessed the prevalence and socio-demographic correlates of bullying in physiotherapy education in Nigeria.

## Methods

Respondents for this cross-sectional study were purposive clinical physiotherapy students from the three out of the six Federal Universities where physical therapists are trained in Nigeria. These institutions are the University of Ibadan (UI), University of Lagos (UNILAG) and the Obafemi Awolowo University (OAU). These institutions are the oldest and foremost training institutions for physiotherapy in Nigeria. In addition, these institutions have longstanding and experienced faculties in the Nigerian context. Respondents in this study were students in the clinical levels (i.e. year three and above) of the current five-year baccalaureate programme in Nigeria. Based on available sample frame of all students in the clinical level of the selected university, a population of 379 was obtained. A sample size formula by Yamane [46] - n = N/1 + N (e^2^) was used to calculated the sample size. Where n is sample size, e is level of error tolerance and N is the population size. Thus, n obtained was 181, however, allowing for 10% non-response, a total of 189 was estimated.

Students Perception of Professor Bullying Questionaire (SPPBQ) was used to assess bullying among the respondents. The SPPBQ contains a working definition of professor/lecturer bullying followed by three questions inquiring about lecturers bullying experiences [47, 48]. The developers of the tool modeled it after earlier tools by Chapell et al. [34] on teacher and professor/instructor/peer bullying experiences. In addition, was the Negative Acts Questionnaire – Revised (NAQ-R) which was designed to assess workplace bullying [5]. The NAQ-R encompassed three underlying factors (personal, work-related and physically intimidating forms of bullying) and also generated a single item measure of bullying [5]. The SPPBQ covers three underlying factors, these are personal characteristics, and academic-related and physically intimidating forms of bullying. Questions on the tool address specific behaviors and answer choices on a Likert scale for frequency, from never to daily. Results of the psychometric evaluation of the tool indicates a Cronbach’s coefficient Alpha that was satisfactory for its various components (academic bullying (α = .901), personal bullying (α = .883), as well as the global component for all 11 items (α = .909). In addition, the criterion validity of the SPPBQ was satisfactory for academic bullying (r = .591, *p* < .001) and personal bullying (r = .289, p < .001) components.

The part of the SPPBQ that assesses professor/instructor bullying experiences was utilized in this study. The aspects of the tool exploring peer bullying experiences were outside the scope of this study. In addition, in place of the demographic aspect of the questionnaire that sought information on personal characteristics, a proforma was designed to obtain context-specific information. The adapted tool was pilot tested among 20 clinical physiotherapy students recruited from the OAU, who were not part of the main study. A test-retest analysis of the global components scores of the tool yielded a Spearmen rho value of 0.969 at *p* = 0.001. Prior to the test-retest survey, a qualitative group debriefing assessment was carried out where each of the items were read out to judge respondents comprehensibility of the items. It was a consensus that the word ‘professor/instructor’ be modified to ‘physiotherapy lecturer’ as it is being commonly used in the study setting. Unlike in some other contexts, where university teachers are referred to as Professors, it is almost insupportable to answer to the name, having not attained the rank. As such, respondents may miss out lecturers of lower ranks in the survey. This survey utilized an in-person, paper and pencil self-administration mode for the collection of data using the questionnaire.

Ethical approval for this study was obtained from the Human Research and Ethics Committee of Institute of Public Health (IPHOAU/12/925), Obafemi Awolowo University, Ile-Ife, Nigeria. Informed consent was obtained from all respondents following full disclosure of the purpose of the study. Respondents were assured of anonymity, as no names or personal identifiers were associated with the data. No teaching faculty was involved in data collection process, so as to limit coercive participation, considering the sensitive nature of the study and also considering that in-person mode of questionnaire administration was used in the study.

### Statistical analysis

Descriptive statistics of mean, standard deviation, frequency and percentages was used to summarize data. Based on responses in section B of the questionnaire, 0, 1, 2, 3, 4 were assigned to ‘never’, ‘now and then’ ‘monthly’, ‘weekly’, and ‘daily’ respectively. Total obtainable score for the 21 questions was 84 with higher scores indicating high level of bullying. Based on the scores, in this study, having a score of 28 or less, 29 to 56, and greater than 56 were categorized by the authors as mild, moderate and severe bullying respectively using percentile cut-points. Pearson Chi-square was used to investigate factors associated with bullying. Alpha level was set at *p* < 0.05. SPSS version 16.0 (Chicago, Illinois: SPSS) was used to analyse data.

## Results

Table 1 shows the socio-demographic characteristics of the respondents in the study. Most of the respondents were single (98.2%) and within the age of 20 to 25 years (94.5%), of Yoruba ethnicity (85.8%), of Christian religion (79.9%) and were of the female gender (51.1%). The result showed that the mean age of respondents was 21.8 ± 1.50. Table 2 shows the frequency distribution on policy and support on bullying. From the result, 37.5 and 42.7% of respondents from Obafemi Awolowo University, Ile-Ife, Nigeria responded in the affirmative (i.e. yes) to having adequate school policy and support on bullying. Responses on having adequate ‘policy’ and ‘support’ on bullying from respondents from University of Ibadan, Nigeria and University of Lagos, Nigeria were 30 and 48%, and 45.2 and 45.2% respectively. In sum, 38.4 and 44.7% of the respondents believed there was adequate school policy and support available on bullying.

**Table 1:** Socio-demographic characteristics of the respondents

Variable	OAU n (%)	UI n (%)	UNILAG n (%)	χ2	*p*-value	All respondents n (%)
Gender						
Male	55 (57.3)	17 (34.0)	35 (47.9)	7.175	0.028	107 (48.9)
Female	41 (42.7)	33 (66.0)	38 (52.1)			112 (51.1)
Age						
> 20	2 (2.08)	6 (12.0)	1 (1.37)			9 (4.11)
20–25	91 (94.8)	44 (88.0)	72 (98.6)	14.067	0.007	207 (94.5)
< 25	3 (3.13)	0 (0.00)	0 (0.00)			3 (1.37)
Ethnicity						
Yoruba	85 (88.5)	44 (88.0)	59 (80.8)			188 (85.8)
Igbo	10 (10.4)	5 (10.0)	12 (16.4)	2.493	0.646	27 (12.3)
Others	1 (1.04)	1 (2.00)	2 (2.74)			4 (1.83)
Religion						
Christianity	83 (86.5)	41 (82.0)	51 (69.9)			175 (79.8)
Islam	6 (6.25)	6 (12.0)	10 (13.7)	8.510	0.075	22 (10.1)
Others	7 (7.29)	3 (6.00)	12 (16.4)			22 (10.1)
Educational Level						
400	51 (53.1)	29 (58.0)	29 (39.7)	4.733	0.094	109 (49.8)
500	45 (46.9)	21 (42.0)	44 (60.3)			110 (50.2)
Marital Status						
Single	94 (97.9)	50 (100)	71 (97.3)	1.305	0.521	215 (98.2)
Married	2 (2.08)	0 (0.00)	2 (2.74)			4 (1.83)

*OAU* Obafemi Awolowo University, *UI* University of Ibadan, *UNILAG* University of Lagos

**Table 2:** Frequency distribution on adequate policy and support on bullying in University

Variable	Respondents Institution					
	OAU n (%)	UI n (%)	UNILAG n (%)	χ2	*p*-value	All respondents n (%)
Policy on bully						
Yes	36 (37.5)	15 (30.0)	33 (45.2)	6.558	0.161	84 (38.5)
No	60 (62.5)	35 (70.0)	40 (54.8)			135 (61.6)
Availability of support on bullying						
Yes	41 (42.7)	24 (48.0)	33 (45.2)	2.220	0.695	98 (44.7)
No	55 (57.3)	26 (52.0)	40 (54.8)			121 (55.3)

*OAU* Obafemi Awolowo University, *UI* University of Ibadan, *UNILAG* University of Lagos

**Fig. 1:**
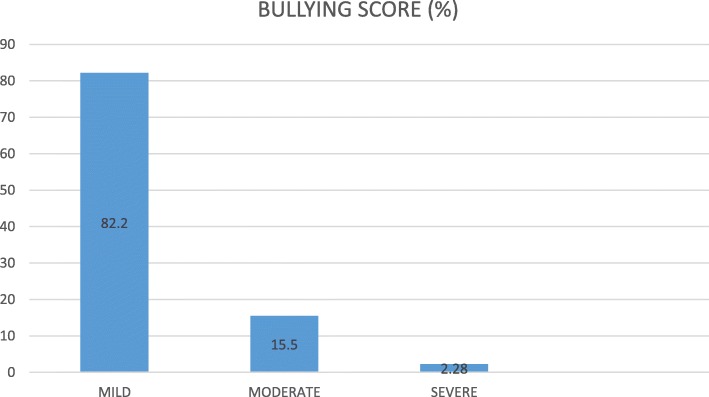
Level of bullying among the respondents

Table 3 shows the distribution of bullying characteristics of the respondents. 94.5% of all respondents had witnessed a physiotherapy student been bullied by a physiotherapy lecturer. 98.6% of all respondents have experienced bullying by a physiotherapy lecturer. 99.5% of all students who had experienced bullying neither stopped or attempted to stop a physiotherapy lecturer from bullying them. 99.1% of the respondents had a recent positive history or experience of bullying in their present level by a physiotherapy lecturer, while none of the respondents (100%) of the respondents stopped or attempted to stop a physiotherapy lecturer from bullying. Table 4 shows the association between lifetime prevalence of bullying and socio-demographic characteristics of the respondents. The result showed that there was no significant association between bullying and each of age (*p* = 0.92), gender (0.07), ethnicity (0.50), religion (0.06), university (0.22), educational level (0.33), marital status (0.064) of the respondents. Table 5 shows the association between points prevalence of bullying and socio-demographic characteristics of the respondents. Similarly, the result showed no significant association between bullying and each of age (0.94), gender (0.97), ethnicity (0.85), religion (0.16), university (0.51), educational level (0.99), and marital status (0.85) of the respondents. Figure 1 shows the total bullying scores of all the respondents. 82.2% has experienced a mild form of bullying, 15.5% has experienced a moderate form of bullying while 2.28% has experienced a severe form of bullying. The results in the figure is from the 98.6% of all respondents that have experienced bullying by a physiotherapy lecturer.

**Table 3:** Frequency distribution on bullying characteristics among the respondents

Item	Respondents Institution					
	OAU n (%)	UI n (%)	UNILAG n (%)	χ2	*p*-value	All respondent n (%)
Ever seen student been bullied?						
Yes	91 (94.8)	47 (94.0)	69 (94.5)	0.040	0.980	207 (94.5)
No	5 (5.21)	3 (6.00)	4 (5.48)			12 (5.48)
Have you ever experienced bullying?						
Yes	95 (99.0)	49 (98.0)	72 (98.6)	0.223	0.894	216 (98.6)
No	1 (1.04)	1 (2.00)	1 (1.37)			3 (1.37)
Attempts by student to stop lecturer bullying?						
Yes	1 (1.04)	0 (0.00)	0 (0.00)	1.287	0.525	1 (0.46)
No	95 (98.9)	50 (100)	73 (100)			218 (99.5)
Have you been bullied in present level?						
Yes	95 (98.9)	49 (98.0)	73 (100)	1.343	0.511	217 (99.1)
No	1 (1.04)	1 (2.00)	0 (0.00)			2 (0.91)
Ever stopped or attempted to stop bullying other students?						
Yes	0 (0.00)	0 (0.00)	0 (0.00)	–	–	0 (0.00)
No	96 (100)	50 (100)	73 (100)			219 (100)

*OAU* Obafemi Awolowo University, *UI* University of Ibadan, *UNILAG* University of Lagos

**Table 4:** Test of association between lifetime prevalence of bullying and socio-demographic characteristics of respondents

Variables	Bullying Response			
	YES n (%)	NO n (%)	χ2	*p*-value
Gender				
Male	104 (47.5)	3 (1.37)	3.184	0.07
Female	112 (51.1)	0 (0.00)		
Age				
< 20	9 (4.11)	0 (0.00)	0.176	0.91
20–25	204 (93.2)	3 (1.37)		
> 25	3 (1.37)	0 (0.00)		
Ethnicity				
Yoruba	185 (84.5)	3 (1.37)	0.502	0.77
Igbo	27 (12.3)	0 (0.00)		
Others	4 (1.83)	0 (0.00)		
Religion				
Christianity	174 (79.5)	1 (0.46)	10.84	0.060
Islam	20 (9.13)	2 (0.91)		
Others	22 (10.1)	0 (0.00)		
University				
OAU	95 (43.4)	1 (0.46)	0.223	0.894
UI	49 (22.4)	1 (0.46)		
UNILAG	72 (32.9)	1 (0.46)		
Level				
400	108 (49.3)	1 (0.46)	0.329	0.566
500	108 (49.3)	2 (0.91)		
Marital Status				
Single	213 (97.3)	2 (0.91)	16.839	0.064
Married	3 (1.37)	1 (0.46)		

*OAU* Obafemi Awolowo University, *UI* University of Ibadan, *UNILAG* University of Lagos

**Table 5:** Test of association between point prevalence of bullying and socio-demographic characteristics of respondents

Variables	Bullying Response			
	YES n (%)	NO n (%)	χ2	*p*-value
Gender				
Male	106 (48.4)	1 (0.46)	0.001	0.974
Female	111 (50.7)	1 (0.46)		
Age				
< 20	9 (4.11)	0 (0.00)	0.117	0.94
20–25	205 (93.6)	2 (0.91)		
> 25	3 (1.37)	0 (0.00)		
Ethnicity				
Yoruba	186 (84.9)	2 (0.91)	0.333	0.847
Igbo	27 (12.3)	0 (0.00)		
Others	4 (1.83)	0 (0.00)		
Religion				
Christianity	174 (79.5)	1 (0.46)	3.636	0.162
Islam	22 (10.1)	0 (0.00)		
Others	4 (1.83)	1 (0.46)		
University				
OAU	95 (43.4)	1 (0.46)	1.343	0.511
UI	49 (22.4)	1 (0.46)		
UNILAG	73 (33.3)	0 (0.00)		
Level				
400	108 (49.3)	1 (0.46)	0.000	0.995
500	109 (49.8)	1 (0.46)		
Marital Status				
Single	213 (97.3)	2 (0.91)	0.038	0.846
Married	4 (1.83)	0 (0.00)		

*OAU* Obafemi Awolowo University, *UI* University of Ibadan, *UNILAG* University of Lagos

## Discussion

This study investigated bullying and its socio-demographic correlates among physiotherapy students in Nigeria. The responding physiotherapy students were mostly females who were within 20 and 25 years. The lifetime prevalence of bullying in this study was 98.6%, while the point prevalence was 99.1%. The high lifetime and point prevalences of bullying observed in this study were comparable to early findings. For example, Clarke and colleagues [49] reported positive history of bullying behaviors (88.72%) among Canadian clinical undergraduates nursing students. Similarly, other international studies have reported up to 90% bullying rates among nursing students in the clinical setting [50–52]. On the other hand, relatively lower rates of bullying were recorded in a Turkish and United Kingdom study that stated only more than half (60, and 53%) of the respondents were exposed to bullying during their education [53, 54]. The higher rates of bullying observed in this study may be an indicator of right abuses that are prevalent in the wider society in Nigeria [55, 56]. According to UNESCO [57] physical and other forms of bullying behaviour in schools’ settings is a reflection of the wider social context [57]. The UNESCO report also suggests that ‘sexual violence and harassment of girls is worse in schools where other forms of violence are prevalent, and in conflict and emergency contexts’ [57]. The recent media reports on the spates of sexual harassment in the academy in Nigeria [58] may just be a pointer to the extent of bullying that have happened and currently going on in the Nigerian comtext. McEvoy [59] has described lecturer bullying to include sexual harassment and hate crimes. Similarly, UNESCO (21) designates sexual violence, including rape and sexual harassment as a form of bullying.

This study findings showed that 100% of the physiotherapy students had experienced bullying behaviours in the clinical settings in forms of being humiliated in connection with their course, being ignored and excluded, spreading of gossip and rumors about them, being shouted at or being the target of spontaneous anger or having an offensive remarks made about them by a physiotherapy lecturer. Consistent with this finding is the report of Foster and Colleagues [51], who identified that 90% of nursing students reported experiencing some form of bullying while on clinical placement. In line with the finding of this study, a report from Turkey revealed that 100% of nursing students in a surveyed reported being yelled or shouted at, or were behaved toward in an inappropriate, nasty, rude or hostile way, or were belittled or humiliated. In this same study, 83.1% of the student nurses reported experiencing academic abuse which included being told negative remarks about becoming a nurse; being assigned responsibilities as punishment rather than for educational purposes; and being punished with poor grades or being shown hostility following an academic accomplishment [50]. Supporting these results, a U.S. study which revealed that the most frequently reported behaviors perceived to be bullying included cursing or swearing (41.1%), inappropriate, nasty, rude or hostile behaviors (41%) and belittling or humiliating behavior (32.7%) [52]. Moreover, Stevenson and colleagues [53] found that the least frequent negative behavior selected by the students was the threat of actual physical violence.

Social norms and power imbalances in schools promote attitudes and practices that tend to subdue students, support unequal gender norms, as well as bear with violence, including corporal punishment [21]. The use of violence to assert discipline and control in the academy is attributable to social norms that support the authority of lecturers over students, and students who defies or choose not to conform to these norms gets punished through violence and bullying [21]. While, discipline is probably the most difficult and unpleasant part of teaching profession [60, 61], however, many traditional approaches to discipline are reported to be negative, punitive and reactive, and results in bad outcomes for all parties involved [62]. Still, corporal punishment is common in Nigeria and is often treated as an integral part of education, holding a place in schools teaching [63] and it is more pronounced in secondary schools than in universities [64, 65]. It is adducible, that bullying in the Nigerian university setting could be as a result of repression or inhibited tendency to implement corporal punishment that is only permissible at lower school level.

The result of this study has also revealed that a large number of physiotherapy students have witnessed an incidence of a physiotherapy lecturer bullying another physiotherapy student and only a whit of the respondents has ever stopped or attempted to stop a physiotherapy lecturer from bullying them or other physiotherapy students in the college. This is consistent with the findings of Clarke and colleagues [49] who stated that students justified not taking action as a result of experiencing bullying behaviors by making excuses for the poor behavior, minimizing the event and its impact, normalizing the behavior and fearing a poor evaluation. Furthermore, Stevenson and colleagues [53] found that students identified that reporting bullying was not worth the effort, wished not to jeopardize their assessment and that it is something that one must simply adapt with. This is also in line with report by Hoel and colleagues [66] who, in a qualitative study investigating the realities and expectations of nursing students, reported that students defended the poor behavior, to the extent of suggesting that it may serve a purpose or that it was due to pressure and/or workload or previous experiences of bullying. In line with the foregoing, it is an anecdote in the study setting that a lot of students seem to be absorbed in the so called ‘culture of silence’ where students out of ignorance or for the fear of intimidation refuses to reports or challenge a harassment or a bullying.

This study further investigated the availability of adequate support and policies on bullying in the various universities. The result revealed that there is no significant difference between bullying and the availability of support and polices. Findings among the few studies that examined associations between policy presence and student bullying were mixed, although more non-significant than significant associations were found. At first glance, one may conclude from these findings that the presence of bullying policies does not influence bullying among students; however, the presence of a policy is necessary but is not sufficient to affect bullying behavior. Indeed, after a policy has been adopted, it must be put into practice. The mere adoption or presence of a policy does not mean that it will be immediately and consistently put into practice exactly as intended [67]. However, the veracity of claims on available of policies to cub or limit bullying in education may at best be speculative, as anecdotal information among the students so indicate.

The result of this also revealed that there was no significant association between bullying and gender, age, ethnicity, religion, university and educational level of the participants in the study. The lack of a significant association between bullying and the socio-demographic characteristics of the students indicates that those involved in bullying do not tend to choose their targets based on the characteristics of the respondents. This finding is in line with Salin’s [68] study indicating that the bullying is enabled by a power imbalance and the low perceived costs of bullying. This study has addressed the prevalence of bullying experiences among clinical physiotherapy students. The study has identified common issues that physiotherapy students face during their education, which leave them feeling powerless and frustrated. A potential limitation of this study may include the non-probability sampling method used, which may impact on the external validity of the findings. Also, the generalizability of the findings is limited to clinical physiotherapy students, especially those from the institutions surveyed. Furthermore, the instrument used in this study was only tested for its reliability and face validity in the pilot study, there is little or no reports on its psychometric properties in many studies nor in this current study’s setting, and this poses a limitation. This is the first empirical report on bullying in physiotherapy education in Nigeria. This report may serve as a precursor for inquiry into bullying in other health professions education in Nigeria. In addition, the reports have implications for necessary policy guidance on addressing bullying and effecting the required change in the organizational culture in the Nigerian health profession educational setting. In addition, the finding of this study may reveal the gap in the extant policy on bullying and the reality. Thus, a qualitative enquiry into the contextual factors engendering bullying in the academy in Nigeria is recommended. Furthermore, there is a need for future studies to examine types and frequencies of academic-related bullying and physical intimidating forms of bullying in the academy.

## Conclusion

There is high lifetime and point prevalence of bullying in physiotherapy education in Nigeria, which are largely unchallenged or redressed. Being a clinical physiotherapy student ordinarily predisposes to bullying without necessary contributions of intrinsic and extrinsic factors. Thus, this study put in empirical perspective, bullying in physiotherapy education in Nigeria, and has the potential to inform policy and practices that may help stem the negative consequences of bullying on a student’s academic performance, as well as social, psychological, and emotional life.

## Data Availability

The datasets obtained and used for analysis in this study are available on reasonable request from the corresponding author.

## Appendix

### Adapted questionnaire from the Student Perception of Professor Bullying Questionnaire (SPPBQ)

The purpose of this confidential questionnaire is to obtain information on student perception of lecturer bullying.

### Section A

After reading the definition below, please answer the following questions about your experiences with bullying. For each question choose an answer as it relates to the frequency on a scale from 0 (Never) to 3 (Very frequently).

**Lecturer Bullying Definition:**

A students is being bullied by a lecturer when he or she uses her/his power to punish, manipulate or belittle the student beyond what would be a reasonable disciplinary procedure by:

1. saying hurtful things to the student (e.g. unfriendly teasing, using a sarcastic haughty manner, using harmful words or names);
2. saying hurtful things about the student’s character or ability (e.g.name calling, yelling, or public ridicule);
3. making obscene gestures to a student;
4. ignoring or neglecting the student;
5. physical actions or attacks that may involve hurting or pushing a student around (e.g. putting tape on a student’s mouth);
6. spreading of gossip or rumors that make other students, lecturers or faculty dislike the student or that get the student into trouble.

**Table:**

S/N		Never	Only once or twice	Occasionally	Frequently
1.	Have you ever seen a physiotherapy student being bullied in university by a physiotherapy lecturer?				
2.	Have you ever been bullied in university by a physiotherapy lecturer?				
3.	Has another physiotherapy student stopped or attempted to stop a physiotherapy lecturer from bullying you?				
4.	In your present level, have you being bullied by a physiotherapy lecturer?				
5.	Have you stopped or attempted to stop a physiotherapy lecturer from bullying other physiotherapy students in college?				

### Section B

The following questions address different components of lecturer bullying as it relates to your experience during the past 6 months.

**Table:**

S/N		Never	Now and then	Monthly	Weekly	Daily
1.	A physiotherapy lecturer withholding information that affects your performance.					
2.	Being humiliated or ridiculed by a physiotherapy lecturer in connection with your course.					
3.	Spreading of gossip and rumors about you by a physiotherapy lecturer.					
4.	Being ignored by a physiotherapy lecturer					
5.	Being excluded by a lecturer that affects your academic performance.					
6.	Having insulting or offensive remarks made about you by a physiotherapy lecturer					
7.	Having insulting or offensive remarks made about your attitudes by a physiotherapy lecturer.					
8.	Crude and offensive sexual remarks directed at you, either publicly or privately by a physiotherapy lecturer.					
9.	Being shouted at or being the target of spontaneous anger by a physiotherapy lecturer.					
10.	Having a physiotherapy lecturer gossip about your sex life or spread rumors about your sexual activities.					
11.	Intimidating behaviors such as finger-pointing, invasion of personal space, shoving, blocking your way by a physiotherapy lecturer.					
12.	Being told or hinted by a physiotherapy lecturer that you are incompetent.					
13.	Repeated reminders of your mistakes by a physiotherapy lecturer.					
14.	Being ignored or facing a hostile reaction when you approach a physiotherapy lecturer.					
15.	Persistent criticism of your mistakes by a physiotherapy Lecturer.					
16.	Having your comments ignored by a Lecturer.					
17.	Having false allegations made against you by a physiotherapy lecturer.					
18.	Being the subject of excessive teasing or sarcasm by a physiotherapy lecturer.					
19.	Threats of violence or physical abuse by a physiotherapy Lecturer.					
20.	Acts of violent or physical abuse by a physiotherapy lecturer.					
21.	Having insulting or offensive remarks made about your private life by a physiotherapy lecturer.					

**Thank you for your time!**

## Abbreviations

OAU: Obafemi Awolowo University
UI: University of Ibadan
UNILAG: University of Lagos
SPPBQ: Students Perception of Professor Bullying Questionnaire
